# Autoinflammatory diseases: a possible cause of thrombosis?

**DOI:** 10.1186/s12959-015-0049-x

**Published:** 2015-05-12

**Authors:** Micaela La Regina, Francesco Orlandini, Raffaele Manna

**Affiliations:** Department of Internal Medicine, Ligurian East Hospital, La Spezia, Italy; Centre of Periodic Fevers – Catholic University of Rome, Rome, Italy

**Keywords:** Autoinflammatory diseases, Familial mediterranean fever, Criopyrinopathies, Pyrinopathies, Periodic fevers, Venous thrombosis, Venous thromboembolism, Arterial thrombosis, Stroke, Myocardial infarction

## Abstract

Autoinflammatory diseases are a group of disorders due to acquired or hereditary disfunction of innate immune system and characterized by systemic or localized manifestations. The prototype is Familial Mediterranean Fever, a monogenic hereditary disorder, whose causing gene (MeFV gene) was identified in 1997 and opened the way to a new fascinanting chapter of rheumatology. A growing body of monogenic and poligenic autoinflammatory disorders has been described since then.

Arterial and venous thrombosis is a common medical problem, with significant morbidity and mortality. Strong evidences from basic research and clinical epidemiological studies support the theory that inflammation and thrombosis can be associated.

Because of their recurrent/chronic inflammatory nature, autoinflammatory diseases are a putative cause of thrombotic manifestations. In the present work, we reviewed the available evidences about monogenic autoinflammatory disorders, complicated by thrombotic manifestations.

## Introduction

Auto-inflammatory diseases (AIDs) are a group of disorders, pathophysiologically characterized by acquired or hereditary defects of innate immune system and clinically by systemic and/or localized, chronic or recurrent inflammatory manifestations. They are a quite recent nosological group. The prototype is Familial Mediterranean Fever, a monogenic hereditary disorder, whose gene causing (MeFV gene) was identified in 1997 and opened the way to a new fascinanting chapter of rheumatology. The discovery of this and other genes, involved in autoinflammatory diseases, indeed, has allowed not only to better understand the single disease, but also the mechanisms of inflammation. The last classification of AIDs distinguishes between monogenic and polygenic disorders. Monogenic diseases can be also distinguished according to pathogenesis or their main clinical features (Table 1) [1].

**Table 1:** **Classification of autoinflammatory diseases and their pathogenesis (modified from Ozen S, Nature Rev Rheumatol 2014; 10:135–147)**

**Monogenic disease**	**Pathogenesis**
**Periodic fever diseases**	
Familial Mediterranean fever, FMF	Defects of IL-1Β family regulation
Hypepr IgD Syndrome, HIDS	Defects of IL-1Β family regulation
TNF-receptor-associated periodic syndrome, TRAPS	Protein mis-folding
Familial cold autoinflammatory syndrome 2, FCAS2	NFk-B activation
Cryopirin-associated periodic syndromes	Defects of IL-1Β family regulation
**Diseases with pyogenic lesions**	
Deficiency of IL-1 receptor antagonist, DIRA	Defects of IL-1Β family regulation
Pyogenic arthritis, pyoderma gangrenosum and acne syndrome, PAPA	Defects of IL-1Β family regulation
Majeed syndrome	Defects of IL-1Β family regulation
**Diseases with granulomatous lesions**	
Blau syndrome	NFk-B activation
**Diseases with psoriasis**	
Deficiency of IL-36 receptor antagonist	Altered IL-36 regulation
**Diseases with panniculitis-induced lipodistrophy**	
Joint contractures, muscle atrophy and panniculitis-induced lipodistrophy syndrome, JMP	Disease linked to the proteasome and/or IFN-γ
Chronic atypical neutrophilic dermatosis with lipodistrophy and elevated temperature, CANDLE	Disease linked to the proteasome and/or IFN-γ
Nakajo-Nishimura syndrome, NNS	Disease linked to the proteasome and/or IFN-γ
**Others**	
PLCγ2-associated antibody deficiency and immune dysregulation syndrome, APLAID	Alteration of inflammation signalling and immune response
**Poligenic diseases**	
Gout	IL-1B activation
Schnitzler syndrome	IL-1B activation
Behçet disease	Spontaneous and/or induced overexpression of pro-inflammatory Th1 type cytokines – HLA-B51
Systemic-onset juvenile idiopathic arthritis (JIA)	IL-1B activation
Crohn’s disease	Altered innate and adaptive immune system (Th1 and Th17 mediated process)
Aphthous stomatitis, pharyngitis and adenitis (PFAPA) syndrome	Altered innate and adaptive immune system (IL-1B–18 activation and Th1 chemokines induction)

Thrombosis is a common medical problem, with significant morbidity and mortality. Chronic inflammatory disorders are recognized risk factors for thrombotic diseases, as inflammatory cytokines modulate the coagulation pathway by enhancing procoagulants and inhibiting anticoagulant and fibrinolytic systems. Further, endothelial dysfunction, another component of the Virchow’s triade leading to thrombosis, is associated with chronic inflammation. Several epidemiological studies reported increased morbidity and mortality from cardiovascular diseases in patients with rheumatoid arthritis [2], and strong evidences from basic research and epidemiological studies support that inflammation and venous thrombosis are associated processes [3].

Because of their recurrent/chronic inflammatory nature, autoinflammatory diseases are a putative cause of thrombotic manifestations. The aim of the present work is to review available evidences about thrombosis and monogenic autoinflammatory disorders. The association with polygenic autoinflammatory disorders will be dealt elsewhere in this issue.

## Review

### Search strategy

We performed a systematic review of studies reporting arterial and venous thrombosis in patients with autoinflammatory diseases. Studies had to have published in peer-reviewed journals.

We searched Medline/PubMed using terms: (((“Thrombosis” [Mesh] OR ((“Superior Vena Cava Syndrome” [Mesh]) OR ((“Infarction, Middle Cerebral Artery” [Mesh]) OR ((“Budd-Chiari Syndrome” [Mesh]) OR ((“Retinal Vein Occlusion” [Mesh]) OR ((“Carotid Artery Thrombosis” [Mesh]) OR ((“Intracranial embolism and thrombosis” [Mesh]) OR ((“Coronary Thrombosis” [Mesh]) OR ((“Sinus Thrombosis, Intracranial” [Mesh]) OR ((“Embolism and Thrombosis” [Mesh]) OR ((“Sagittal Sinus Thrombosis” [Mesh]) OR ((“Cavernous Sinus Thrombosis” [Mesh]) OR ((“Lateral Sinus Thrombosis” [Mesh]) OR ((“Venous Thrombosis” [Mesh]) OR ((“Intracranial Thrombosis” [Mesh]) OR ((“Upper Extremities Deep vein Thrombosis” [Mesh]) OR ((“Stroke”) OR ((“Myocardial Infarction”) OR ((“Deep vein thrombosis”) AND ((“Hereditary Autoinflammatory Disorders” [Mesh]) retrieving 41 articles. Eighteen were eligible for the review. Then, we used the same search terms AND ((“Familial Mediterranean Fever”), retrieving 33 papers, 22 elegible; AND ((“Periodic fever, familial, autosomal dominant” [Supplementary Concept] [Mesh]) AND ((“Periodic fever, familial autosomal dominant” [All Fields] [Mesh]), AND ((“Cryopyrin-Associated Periodic Syndromes”) AND ((“Familial Cold Autoinflammatory Syndrome 2” [Supplementary Concept]) AND ((“Inflammasomes”) AND ((“NLRP3 protein, human” [Supplementary Concept] [Mesh]) AND ((“Pyogenic arthritis, pyoderma gangrenosum and acne syndrome”) AND ((“Deficiency of IL-36 receptor antagonist”) AND ((“Joint contractures, muscle atrophy and panniculitis-induced lipodistrophy syndrome”) AND ((“Chronic atypical neutrophilic dermatosis with lipodistrophy and elevated temperature”) AND ((“PLCγ2-associated antibody deficiency and immune dysregulation syndrome”) AND ((“NakaJo-Nishimura syndrome”) with no items found; AND ((“Deficiency of IL-1 receptor antagonist”) AND ((“Majeed syndrome”) with 7 items found, no one elegible for our review; AND ((“Muckle-Wells”) with 2 items found, one eligible for our review.

Eligibility criteria were: studies reporting cases of autoinflammatory syndromes complicated by arterial or venous thrombotic manifestations, studies investigating coagulation in autoinflammatory syndromes or autoinflammatory genes mutations in patients with cardiovascular diseases.

## Results

Our research retrieved 33 papers about familial Mediterranean fever (FMF) complicated by thrombotic diseases (22 after exclusion of duplicated papers), 2 about TNF receptor associated periodic syndrome (TRAPS) and increased risk of atherosclerosis and acute myocardial infarction and one about Muckle-Wells syndrome (MWS)/neonatal-onset multisystem inflammatory disease (NOMID) overlap complicated by cerebrovascular accident (CVA).

This latter reports the case of a woman with overlapping manifestation of MWS and NOMID that experienced a cerebrovascular attack (leftsided weakness, vertigo and paresthesia; altered brain CT scan) at the age of 28. The authors do not mention other traditional risk factors for thrombotic diseases; anyway, they specify they did not search for anticardiolipin antibodies.

The association of FMF with thrombotic manifestations, instead, relies on 4 laboratory studies, 2 review, 9 case reports and 9 case series, ranging from pulmonary embolism to thrombosis in atypical sites, stroke or myocardial infarction. Details are shown in Table 2.

**Table 2:** **List of case reports and case series about thrombotic manifestations in FMF patients**

**Authors**	**Type of publication**	**Thrombotic manifestation**	**Other thrombosis risk factors**	**Genetically proven FMF**
Joo K et al.	Case report	total thrombosis of splenic vein with partial thrombosis of proximal superior mesenteric vein, main portal vein and intrahepatic both portal vein	Decreased protein S activity	3 mutations (p.Glu148Gln,p.Pro369Ser, p.Arg408Gln)
Ambartsymian SV	Autoptic case series (68 patients)	amyloid angiopathias, with narrowing or obstructing of arterioles and coronary vasculitis	Data not available	Data not available
Luger S et al.	Case report	Brain stem infarction due to CNS vasculitis	Data not available	Yes
Feld O et al.	Review and case-series (11 patients)	Stroke/TIA	renal amyloidosis and haemodialysis, and three had smoking, hypertension, diabetes mellitus and obesity	Data not available
Unal F et al.	Case series (2 patients)	Budd-Chiari syndrome	Data not available	Yes
Korkmaz C et al.	Review and case series (2 patients)	1. Left cerebral infarct 2. Suspected mesenteric ischemia	Amyloidosis, anticardiolipin antibodies Pregnancy	Yes Homozigosity for M694V Data not available
Ruiz XD et al.	Case report	Pulmonary embolism	Data not available	Heterozygous M649V
Standing AS et al.	Case report	Budd-Chiari syndrome	Heterozygous for MTHFR mutation	Homozygous E148Q
Kalyonku U et al.	Case series (7 patients)	Cerebrovascular accident	In 3 patients: significant patent foramen ovale, thalassaemia major and carotid artery dissection	Yes All homozygosis for MEFV mutations (6/7 homozygosis for M694V)
Sari S et al.	Case series (2 patients)	Budd-Chiari syndrome	FV Leiden single mutation Homozygosity for MTHFR mutation	Yes Homozygosity for M694V
Aoun G et al.	Case report	Stroke (right lateral medullary syndrome)	Lupus anticoagulant FV leiden and MTHFR heterozygous mutations	Yes Homozygosity for M694V
Uyarel U et al.	Case report	Acute myocardial infarction	No known coronary risk factors	Data not available
Serrano R et al.	Case report	Acute myocardial infarction due to coronary vasculitis	Amyloidosis Chronic renal failure on hemodyalisis Cyclosporin	Data not available
Ustundag Y et al.	Case report	Superior vena cava thrombosis	Obesity	Not available
Finsterer J et al.	Case report	CNS and PNS vasculitis or amyloidosis	Data not available	Yes Homozygosity for M694V
Reuben A et al.	Case series (7 patients)	Renal vein thrombosis	Amyloidosis	Data not available
Puricel S et al.	Case series (1 patient)	Acute coronary syndrome	Data not available	Data not available
Lidar M et al.	Case series (6 patients)	Thrombo-embolism	Data not available	Data not available

A series of patients with premature myocardial infarction and a series of patients with symptoms suggestive of TRAPS support the relationship between atherosclerosis and TNFR-1 mutations.

### Familial mediterranean fever and stroke

The occurrence of cerebral stroke in FMF patients is limited to three cases and two retrospective studies [4-8].

One case report is about a child, who in addition to FMF had several prothrombotic defects [4], the second about a 21 year-old man with nephrotic syndrome complicated by AA amyloidosis, and lupus anticoagulant in low titre [5], the third about a young patient who suffered of a brain stem infarction during a typical FMF attack [6]. All of them have genetically proven FMF.

The retrospective study by Kalyoncu et al. [7] identified seven cases of stroke among 3034 FMF patients. They were younger (mean age 28.5 years) compared to patients with stroke but not FMF, and vertebrobasilar vessels were the most affected. All the patients were homozygous for the MEFV mutations (M694V in six patients), but they started colchicine late, so they were exposed to long-lasting inflammation. Three of them had also established risk factors for stroke, such as haemodynamically significant patent foramen ovale, thalassaemia major and carotid artery dissection.

The second retrospective study by Feld et al. [8] reported a diagnosis of stroke/transient ischaemic attack (TIA) in 11 FMF patients younger than 50 years, admitted to Sheba Medical Center from 1985 to 2010. Also in this series, other risk factors prevailed: two patients had renal amyloidosis and were on haemodialysis, and three had at least one traditional risk factor such as smoking, hypertension, diabetes mellitus and obesity.

Being FMF characterized by massive inflammation, either during attacks and free intervals, FMF patients should display an increased rate of atherosclerotic vascular disease, including cerebral stroke. Some studies, indeed, reported an increased intima-media thickness of the carotid artery and other markers of enhanced atherosclerosis in FMF [9-13]. Not to forget also amyloidosis - the most dangerous FMF-complication, which is characterized by a thrombophilic profile, with an increased frequency of venous and arterial thrombosis - and vasculitis, another process responsible for stroke in FMF patients. Henoch-Shoenlein Purpura (HSP), Polyarteritis nodosa (PAN) and Behcet’s disease (BD), common vasculites in FMF, can affect central nervous system [14,15]: BD more than HSP more than PAN [16-19]. Sinus vein thrombosis, stroke, multifocal neurologic dysfunctions of cranial nerves and retinal vessel occlusion have been described in patients with BD and FMF [20].

### Familial Mediterranean fever and myocardial infarction

We found 2 case reports of myocardial infarction occurring in patients with FMF [21,22]. A third case is included in a study on aetiology of acute corornary syndrome under the age of 30 [23]. The patient reported by Urayel et al. is a 22-year-old man who developed a fatal myocardial infarction after stopping colchicine [21]. The other one by Serrano et al. is a 29-year-old Spanish woman with a history of FMF, AA amyloidosis and previous renal transplantation who experienced a fatal acute myocardial infarction, due to coronary vasculitis with hystopathologic features different from PAN [22].

The review by Rigante et al. about cardiovascular manifestations in autoinflammatory diseases [24] cites the work by Grimaldi et al. where a MEFV mutation (M694V) was significantly overrepresented in a cohort of infarcted patients from Sicily than in age-matched healthy controls and continued to predict a significant risk to develop MI, even after adjustment for well-known MI risk factors [25].

At the end, there is an autoptic study including sixty-eight patients with FMF and cardiac lesions that shed light on the mechanisms responsible for myocardial infarction in FMF population. The author described amyloid angiopathias, which were more expressed in arteriolar walls, with narrowing or obstruction of lumina, associated with coronary vasculitis. Other predisposing pathogenic factors can be atherosclerotic changes of the vessels, complicated by amyloid depositions in the vessels walls [26].

### Familial mediterranean fever and venous thrombosis in typical and atypical site

No cases of lower or upper extremities deep vein thrombosis in patients with FMF are described in literature. There is just the case of a 43-year-old Turkish man with FMF presenting with pulmonary embolism [27] and a case series of 6 patients with FMF and amyloidosis who died due to thromboembolic phoenomena (one massive pulmonary embolism) [28].

Among atypical sites, splancnic district appears the most affected. We found 4 case reports of Budd-Chiari syndrome [29-31] and 1 case of portal, splenic and superior mesenteric vein thrombosis [32] occurring in patients affected by FMF. There is also another case where a mesenteric thrombosis was suspected but it can not be ascertained because the relatives refused autopsy [5].

Superior vena cava thrombosis complicating FMF was described in a woman who developed also obstructive sleep apnea because of thrombosis-induced soft tissue edema in the upper airways [33].

Renal vein thrombosis, instead, was described in 7 of 57 FMF patients with nephrotic syndrome, histologically proven amyloidosis, and progressive renal failure [34].

### Laboratory studies

Our search identified also four studies and one review investigating coagulation parameters in FMF patients.

Aksu et al. demonstrated the presence of a hypercoagulable state in 27 attack-free FMF patients without amyloidosis [35]. They found shortened TT and prothrombin time, decreased protein C activity and increased levels of human prothrombin fragment F 1 + 2 in FMF patients compared to healthy controls, in absence of other predisposing factors for thrombosis.

Demirel et al. investigated thrombotic and fibrinolytic markers in 64 FMF patients during and outsides attacks and in healthy controls [36]. They found a prolongation of PT during attacks, increased levels of PAI-1, inhibitor of fibrinolysis and maker of endotelial dysfunction compared to attack-free patients and healthy controls, but also increased levels of tissue plasminogen activator (tPA), trigger of the fibrinolytic system, and decreased levels of P-selectin, marker of thrombocyte activation, during the attack period compared to healthy controls. The levels of tPA were found high also in the attack-free period compared to the control group, but the difference was not statistically significat. vW factor and F VIII levels were comparable with those of the controls. At the end the authors hypothesize that hypercoagulability may have a role in the etiopathogenesis of FMF and PAI-1 could be used as a marker of the attacks.

Ertenli et al. studied fibronectin (FN) and thrombospondin (TSP) that just play a role in coagulation and inflammation. Plasma fibronectin and thrombospondin levels during FMF attack periods resulted significantly higher than after attacks resolution. A significant correlation between both FN, TSP and CRP levels and WBC counts was also reported [37].

Gasparyan et al. reviewed the evidences about the use of mean platelet volume (MPV), a parameter readily available from blood counters, as reflection of prothrombotic and pro-inflammatory situations [38]. He found that FMF attacks are associated with a decrease of MPV in pediatric patients [39], probably due to the consumption of large platelets at the sites of serositis. In attack-free adult FMF patients, instead, circulating platelets tend to be larger and it may be considered as a marker of cardiovascular risk [40].

At the end, Bavbek N et al. [41] demonstrated a high level of Thrombin-activatable fibrinolysis inhibitor (TAFI) antigen in attack-free period of FMF disease, supporting the idea of hypercoagulability.

### Tumor necrosis factor receptor and associated periodic syndrome (TRAPS) and myocardial infarction

Polymorphism R92Q of the tumor necrosis factor receptor 1 (TNFR-1) gene has been associated with myocardial infarction (MI) in a cohort of 95 individuals with premature myocardial infarction and familiarity for MI. The population-adjusted odds ratio (OR) for MI associated with allele Q carrying was 2.15 (95% CI: 1.09-4.23). To search for its responsibility in atherosclerosis, this polymorphism was also genotyped in the AXA Study (ultrasound examinations of carotid and femoral arteries in the context of an employment medical examination, 733 subjects), the EVA Study (ultrasound examinations of carotid arteries in a study of cognitive and vascular ageing, 1092 subjects) and the GENIC Study [on brain infarction (BI), 912 subjects]. In the AXA Study, a positive association with a carotid plaque was found among smokers, carrying the 92Q allele (OR 5.07; 95% CI: 1.64-15.63) and with a thickening of the carotid intima-media thickness (IMT) (0.59 (0.11) vs 0.54 (0.11), P = 0.045). In the EVA Study, carriers of allele 92Q had an increased mean carotid IMT 0.70 (0.09) vs 0.67 (0.13), P = 0.02). No significant association of the 92Q allele was found with BI in the GENIC Study [42]. Some years later Stojanov S et al. postulated that the TNFRSF1A V173D cleavage site mutation may be associated with an increased risk for cardiovascular complications and showed a strong response to etanercept [43].

The link between TRAPS and atherosclerosis can be mediated also by adipokines, as it has been demonstrated on one hand, that serum leptin significantly correlates with markers of subclinical atherosclerosis [44] and elevated adiponectin with a higher risk of cardiovascular disease [45] and, on the other hand, preliminary research showed a correlation between serum leptin and adiponektin and disease activity and/or severity [46].

## Discussion

Inflammation is the physiologic response of the body to various insults. Generally, it is self-limited and specific to injury. Sometimes the inflammatory response is inappropriate in terms of triggers (self, inapparent or otherwise innocuous), intensity or duration. Excessive inflammation due to hereditary defects in inflammatory cells occurs in the autoinflammatory disorders, topic of the present review.

Similarly, haemostasis is the normal response of vessels to injury by forming a clot that serves to limit haemorrhage. Thrombosis, instead, is a pathological clot formation that results when hemostasis is excessively activated in absence of bleeding.

Inflammation and coagulation are intricated processes that show crosstalk at numerous levels. Inflammation induces and/or enhances thrombosis and viceversa.

If we look at the inflammatory pathway of hereditary autoinflammatory diseases, it just displays some crosstalk between inflammation and coagulation. Mutated pyrin and cryopyrin, isoprenoids and their products and mutated TNFR1 enhance intracellular enzymes such caspases, transcriptional factors such as NFkB and cytokines like IL-1B, known to increase the release of tissue plasminogen activator (tPA) and urokinase-type plasminogen activator (uPA) from the storage sites in vascular endothelial cells, a sustained and late release of PAI-1 and downregulate thrombomodulin and subsequently the activation of protein C, a natural anticoagulant. Neverthless, autoinflammation can lead to thrombosis, via endothelial injury and endothelial cell dysfunction (ECD), by the activation of platelets, leukocytes and endothelial cells or via Tissue factor (TF) overexpression, in absence of vessel wall damage [47].

On the other hand, coagulation can modulate inflammation, as

- thrombin and some other coagulation factors may bind to protease-activated receptors (PAR) on endothelial and mononuclear cells, fibroblasts, platelets and smooth muscle cells with subsequent production of growth factors and cytokines including IL-6 and IL-8 [48]
- thrombin may induce endothelium to express P-selectin and to secrete palsminogen activating factor (PAF) and work as a chemotactic factor for leukocytes [49,50]
- fibrinogen and fibrin can directly stimulate mononuclear cells to secrete proinflammatory molecules, such as TNF-, IL-1, and MCP-1 [50]
- platelets can activate dendritic cells through which they may regulate T- and B- cell responses, and along with platelet microparticles, activate complement cascade [51]
- platelet derived chemokines can play important roles in innate immunity [52,53].

With these assumptions, we would have expected much more cases of thrombosis in patients affected by autoinflammatory diseases than we found, and not only in patients affected by familial mediterranean fever.

Here, we would try to give some explanations.

First of all, hereditary autoinflammatory diseases (HAID) are rare diseases, so the small prevalence of thrombosis could be only apparent. In fact, the seven cases of stroke out of 3034 patients in the series reported by Kalyoncu et al. [54] correspond to a prevalence higher than general population (0.2 vs. 0.005–0.01% for adults <50 year old). The same is for the Budd-Chiari syndrome whose prevalence ranges from 1.4-2.4/million inhabitants and we found 4 cases complicating FMF that affects approximately 100.000-150.000 individuals worlwide [55].

Second, autoinflammatory diseases other than FMF are much rarer, so the lack of case reports can not exclude the existence of complicating thromboses.

Third, the duration of inflammation can play a role in the prevalence of thrombosis in the different autoinflammatory disorders. In this view, an autoinflammatory disease, like FMF, characterized by intermittent and short-lasting inflammatory attacks could be less frequently complicated by thrombosis, while the persisting inflammation of Behcet’s disease, can justify its high frequency of thrombotic complications. Just considering fibrinogen kinectics, short-lasting inflammation is less likely to determine thrombosis [56]. In this view, FMF patients with more severe mutations, such as M694V, associated with a higher frequency of attacks could experience thrombosis more often. Furthermore, a direct mutation effect can not be excluded, as more than one study has demonstrated that M694V is associated to vascular involvement in Behcet’s diseases [20,57], even if not in all the ethnic groups [58,59]. So, the high prevalence of M69V mutation in the reported cases of FMF complicated by thrombosis could not be a chance.

Fourth, the reported cases are all symptomatic. We miss data about asymptomatic thrombosis complicating HAIDs to support autoinflammation as a cause of thrombosis.

Lastly, basic studies reported an activation of both coagulation and fibrinolysis in FMF patients, suggesting a sort of balance between the two processes during and out of the attacks and clinical studies disclosed the presence of additional thrombotic risk factors, first of all secondary amyloidosis, in all the patients, but one. So, it is conceivable that additional thrombotic risk factors are necessary to break this balance between inflammation-induced coagulation and fibrinolysis and develop thrombosis in FMF patients. A recent cross-sectional study has shown an increased risk of cardiovascular disease in Turkish FMF patients with nephrotic-range proteinuria and amyloidosis compared with patients with nondiabetic glomerulopathy [60].

Another important issue to be addressed is the role of colchicine treatment in preventing thrombosis. By suppressing inflammation and preventing amyloid deposistion, colchicine offers at least two ways to avoid thrombotic complications. Indeed, most of the patients in the case reports developed thrombotic complications before starting or after stopping colchicine. A retrospective investigation about the prevalence of ischemic heart disease and cardiovascular risk factors in FMF has shown that colchicine therapy ameliorates cardiovascular risk profile and makes FMF patients comparable to healthy controls [61]. Similarly, some case–control studies including FMF patients on colchicine therapy did not display significant differences in terms of subclinical atherosclerosis between FMF patients and healthy subjects [62]. The ongoing Canakinumab Anti-inflammatory Thrombosis Outcomes Study (CANTOS) is just testing whether reducing inflammation among men and women who have had a prior heart attack and have CRP > 2 ngm/ml can reduce the risk of another cardiovascular event happening in the future. If positive, CANTOS will provide the first cytokine-based therapy for the secondary prevention of cardiovascular disease and new-onset diabetes [63].

## Conclusions

If autoinflammatory diseases are a possible cause of thrombosis is still to be stated.

At present, a higher prevalence of thrombotic manifestations compared to general population and a concomitant activation of inflammation and coagulation cascade have been demonstrated in FMF patients.

In the next future, the existing international registries, including patients with any of the HAIDs, could try to address the issue of prevalence. Large randomized controlled studies, instead, could clarify the role of colchicine/anti-TNF/anti-IL-1B treatment or assess the responsibility of particular mutations. Once the thrombotic face of autoinflammatory diseases will be displayed, the need of thromboprophylaxis during attack periods or the screening for thrombophilia in HAIDs patients will be emerging issues.

## References

[1] Ozen S, Bilginer Y (2014). A clinical guide to autoinflammatory diseases: familial Mediterranean fever and next-of-kin. Nature Rev Rheumatol.

[2] Ungprasert P, Srivali N, Spanuchart I, Thongprayoon C, Knight LE (2014). Risk of venous thromboembolism in patients with rheumatoid arthritis: a systemic review and metanalysis. Clin Rheumatol.

[3] Tichelaar YIG, Kluin-Nelemans HJC, Meijer K (2012). Infections and inflammatory disease as risk factors for venous thrombosis. A systematic review. Thromb and Haemost.

[4] Aoun EG, Musallam KM, Uthman I, Beydoun A, El-Haji T, Taher AT (2009). Childhood stroke in a child with familial Mediterranean fever carrying several prothrombotic risk factors. Lupus.

[5] Korkmaz C, Kasifoglu T, Bilge NSY, Gonullu E, Oge T (2013). Is there a thrombotic tendency in patients with familial Mediterranean fever? A small case series and review of the literature. Ann Paediatr Rheum.

[6] Luger S, Harter PN, Mittelbronn M, Wagner M, Foerch C (2013). Brain stem infarction associated with familial Mediterranean fever and central nervous system vasculitis. Clin Exp Rheumatol.

[7] Kalyoncu U, Eker A, Oguz KK, Kurne A, Kalan I, Topcuoglu AM (2010). Familial Mediterranean fever and central nervous system involvement: a case series. Medicine (Baltimore).

[8] Feld O, Yahalom G, Livneh A (2012). Neurologic and other systemic manifestations in FMF: published and own experience. Best Pract Res Clin Rheumatol.

[9] Akdogan A, Calguneri M, Yavuz B, Arslan EB, Kalyoncu U, Sahiner L (2006). Are familial Mediterranean fever (FMF) patients at increased risk for atherosclerosis? Impaired endothelial function and increased intima media thickness are found in FMF. J Am Coll Cardiol.

[10] Terekeci HM, Oktenli C, Ozgurtas T, Nalbant S, Top C, Celik S (2008). Increased asymmetric dimethylarginine levels in young men with familial Mediterranean fever (FMF): is it early evidence of interaction between inflammation and endothelial dysfunction in FMF?. J Rheumatol.

[11] Tavil Y, Ozturk MA, Ureten K, Sen N, Kaya MG, Cemri M (2008). Assessment of aortic wall stiffness in patients with Familial Mediterranean Fever. Joint Bone Spine.

[12] Caliskan M, Gullu H, Yilmaz S, Erdogan D, Unler GK, Ciftci O (2007). Impaired coronary microvascular function in familial Mediterranean fever. Atherosclerosis.

[13] Yildiz M, Masatlioglu S, Seymen P, Aytac E, Sahin B, Seymen HO (2006). The carotid-femoral (aortic) pulse wave velocity as a marker of arterial stiffness in familial Mediterranean fever. Can J Cardiol.

[14] Schwartz T, Langevitz P, Zemer D, Gazit E, Pras M, Livneh A (2000). Behcet’s disease in Familial Mediterranean fever: char- acterization of the association between the two diseases. Semin Arthritis Rheum.

[15] Ozdogan H, Arisoy N, Kasapcapur O, Sever L, Caliskan S, Tuzuner N (1997). Vasculitis in familial Mediterranean fever. J Rheumatol.

[16] Ozkaya O, Bek K, Alaca N, Ceyhan M, Acikgoz Y, Tasdemir HA (2007). Cerebral vasculitis in a child with Henoch–Schonlein purpura and familial Mediterranean fever. Clin Rheumatol.

[17] Akman-Demir G, Gul A, Gurol E, Ozdogan H, Bahar S, Oge AE (2006). Inflammatory/demyelinating central nervous system involvement in familial Mediterranean fever (FMF): coincidence or association?. J Neurol.

[18] Glikson M, Galun E, Schlesinger M, Cohen D, Haskell L, Rubinow A (1989). Polyarteritis nodosa and familial Mediter- ranean fever: a report of 2 cases and review of the literature. J Rheumatol.

[19] Tinaztepe K, Gucer S, Bakkaloglu A, Tinaztepe B (1997). Familial Mediterranean fever and polyarteritis nodosa: experience of five paediatric cases. A causal relationship or coincidence?. Eur J Pediatr.

[20] Rabinovich E, Shinar Y, Leiba M, Ehrenfeld M, Langevitz P, Livneh A (2007). Common FMF alleles may predispose to development of Behcet’s disease with increased risk for venous thrombosis. Scand J Rheumatol.

[21] Uyarel H, Karabulut A, Okmen E, Cam N (2006). Familial Mediterranean fever and acute anterior myocardial infarction in a young patient. Anadolu Kardiyol Derg.

[22] Serrano R, Martínez MA, Andrés A, Morales JM, Samartin R (1998). Familial Mediterranean fever and acute myocardial infarction secondary to coronary vasculitis. Histopathology.

[23] Puricel S, Lehner C, Oberhänsli M, Rutz T, Togni M, Stadelmann M (2013). Acute coronary syndromes in patients younger than 30 years – aetiologies, baseline characteristics and long-term clinical outcome. Swiss Med Wkly.

[24] Rigante D, Cantarini L, Imazio M, Lucherini OM, Sacco E, Galeazzi M (2011). Autoinflammatory diseases and cardiovascular manifestations. Ann Med.

[25] Grimaldi MP, Candore G, Vasto S, Caruso M, Caimi G, Hoffmann E (2006). Role of the pyrin M694V (A2080G) allele in acute myocardial infarction and longevity: a study in the Sicilian population. J Leukoc Biol.

[26] Ambartsymian SV (2012). Myocardial infarction in patients with familial Mediterranean fever and cardiac lesions. Georgian Med News.

[27] Ruiz XD, Gadea CM (2011). Familial Mediterranean fever presenting as pulmonary embolism. Conn Med.

[28] Lidar M, Pras M, Langevitz P, Livneh A (2002). Thoracic and lung involvement in familial Mediterranean fever. Clin Chest Med.

[29] Standing AS, Eleftheriou D, Lachmann HJ, Brogan PA (2011). Familial Mediterranean fever caused by homozygous E148Q mutation complicated by Budd-Chiari syndrome and polyarteritis nodosa. Rheumatology (Oxford).

[30] Sari S, Egritas O, Bukulmez A, Dalgic B, Soylemezoglu O (2009). Is familial Mediterranean fever a possible cofactor for Budd-Chiari syndrome?. J Pediatr Gastroenterol Nutr.

[31] Unal F, Cakir M, Baran M, Arıkan C, Yuksekkaya HA, Aydoğdu S (2012). Liver involvement in children with Familial Mediterranean fever. Dig Liver Dis.

[32] Joo K, Park W, Chung MH, Lim MJ, Jung KH, Heo Y (2013). Extensive thrombosis in a patient with familial Mediterranean fever, despite hyperimmunoglobulinemia D state in serum. J Korean Med Sci.

[33] Ustündag Y, Bayraktar Y, Emri S (1998). Superior vena cava thrombosis and obstructive sleep apnea in a patient with familial Mediterranean fever. Am J Med Sci.

[34] Reuben A, Hirsch M, Berlyne GM (1977). Renal vein thrombosis as the major cause of renal failure in familial Mediterranean fever. Q J Med.

[35] Aksu G, Can O, Kaan K, Ferah G, Necil K (2007). Hypercoagulability: interaction between inflammation and coagulation in familial Mediterranean fever. Clin Rheumatol.

[36] Demirel A, Celkan T, Kasapcopur O, Bilgen H, Ozkan A, Apak H (2008). Is Familial Mediterranean Fever a thrombotic disease or not?. Eur J Pediatr.

[37] Ertenli I, Kiraz S, Oztürk MA, Haznedaroglu IC, Celik I, Kirazli S (2001). Plasma fibronectin- and thrombospondin-adhesive molecules during acute attacks and attack-free periods of familial Mediterranean fever. Rheumatol Int.

[38] Gasparyan AY, Ayvazyan L, Mikhailidis DP, Kitas GD (2011). Mean platelet volume: a link between thrombosis and inflammation?. Curr Pharm Des.

[39] Makay B, Türkyilmaz Z, Unsal E (2009). Mean platelet volume in children with familial Mediterranean fever. Clin Rheumatol.

[40] Coban E, Adanir H (2008). Platelet activation in patients with familial mediterranean fever. Platelets.

[41] Bavbek N, Ceri M, Akdeniz D, Kargili A, Duranay M, Erdemli K (2014). Higher thrombin activatable fibrinolysis inhibitor levels are associated with inflammation in attack-free familial Mediterranean fever patients. Ren Fail.

[42] Poirier O, Nicaud V, Gariépy J, Courbon D, Elbaz A, Morrison C (2004). Polymorphism R92Q of the tumour necrosis factor receptor 1 gene is associated with myocardial infarction and carotid intima- media thickness - the ECTIM, AXA, EVA and GENIC Studies. Eur J Hum Genet.

[43] Stojanov S, Dejaco C, Lohse P, Huss K, Duftner C, Belohradsky BH (2008). Clinical and functional characterisation of a novel TNFRSF1A c.605T>A/V173D cleavage site mutation associated with tumour necrosis factor receptor-associated periodic fever syndrome (TRAPS), cardiovascular complications and excellent response to etanercept treatment. Ann Rheum Dis.

[44] Beltowski J (2006). Leptin and atherosclerosis. Atherosclerosis.

[45] Wannamethee SG, Welsh P, Whincup PH, Sattar N (2011). High adiponectin and increased risk of cardiovascular disease and mortality in asymptomatic older men: does NT-proBNP help to explain this association?. Eur J Cardiovasc Prev Rehabil.

[46] Cantarini L, Obici L, Simonini G, Cimaz R, Bacarelli MR, Merlini G (2012). Serum leptin, resistin, visfatin and adiponectin levels in tumor necrosis factor receptor-associated periodic syndrome (TRAPS). Clin Exp Rheumatol.

[47] Poredos P, Jezovnik MK (2007). The role of inflammation in venous thromboembolism and the link between arterial and venous thrombosis. Int Angiol.

[48] Coughlin SR (2000). Thrombin signaling and protease-activated receptors. Nature.

[49] van der Poll T, de Jonge E, Levi M (2001). Regulatory role of cytokines in disseminated intravascular coagulation. Semin Thromb Hemost.

[50] Szaba FM, Smiley ST (2002). Roles for thrombin and fibrin (ogen) in cytokine/chemokine production and macrophage adhesion in vivo. Blood.

[51] Peerschke EIB, Yin W, Ghebrehiwet B (2008). Platelet mediated complement activation. Adv Exp Med Biol.

[52] Levi M, van der Poll T (2010). Inflammation and coagulation. Crit Care Med.

[53] Faioni EM, Cattaneo M (2011). Inflammation and thrombosis-brothers in arms. Eur Oncol Haematol.

[54] Kalyoncu U, Eker A, Oguz KK, Kurme A, Kalan I, Topcuoglu AM (2010). Familial Mediterranean fever and central nervous system involvement: a case series. Medicine.

[55] Ben-Chetrit E, Touitou I (2009). Familial Mediterranean fever in the world. Arthritis Rheum.

[56] de Moerloose P, Boehlen F, Neerman-Arbez M (2010). Fibrinogen and the risk of thrombosis. Semin Thromb Hemost.

[57] Atagunuz P, Ergun T, Direskeneli H (2003). MEFV mutations are increased in Behçet’s disease (BD) and are associated with vascular involvement. Clin Exp Rheumatol.

[58] Yazici A, Cefle A, Savli H (2012). The prevalence of MEFV gene mutations in behcet’s disease and their relation with clinical findings. Rheumatol Int.

[59] Ben-Chetrit E, Cohen R, Chajek-Shaul T (2002). Familial mediterranean fever and Behçet’s disease–are they associated?. J Rheumatol.

[60] Yilmaz MI, Demirkaya E, Acikel C, Saldir M, Akar S, Cayci T, et al. Endothelial function in patients with familial Mediterranean fever-related amyloidosis and association with cardiovascular events. Rheumatology (Oxford). 2014. [Epub ahead of print]. 10.1093/rheumatology/keu231 24907154

[61] Langevitz P, Livneh A, Neumann L, Buskila D, Shemer J, Amolsky D (2001). Prevalence of ischemic heart disease in patients with familial Mediterranean fever. Isr Med Assoc J.

[62] Sari I, Karaoglu O, Can G, Akar S, Gulcu A, Birlik M (2007). Early ultrasonographic markers of atherosclerosis in patients with familial Mediterranean fever. Clin Rheumatol.

[63] Ridker PM, Thuren T, Zalewski A, Libby P (2011). Interleukin-1β inhibition and the prevention of recurrent cardiovascular events: rationale and design of the Canakinumab Anti-inflammatory Thrombosis Outcomes Study (CANTOS). Am Heart J.

